# 

**Published:** 1869-03-15

**Authors:** 


### CHICAGO MEDICAL JOURNAL.

J. ADAMS ALLEN, M.D., LL.D., Editor. WALTER HAY, A.M., M.D., Associate Editor.

All letters for THE JOURNAL should be addressed to DR. J. ADAMS ALLEN, No. 71 Dearborn Street, Chicago, Ill.

TERMS—$3 PER ANNUM, IN ADVANCE.

$4 if not paid before 1st of July, in each year. Single Copies, 25 Cents.

### *CONTENTS.*

ORIGINAL COMMUNICATIONS:

Analysis of Hot Springs, Arkansas ... 161

Cases of Malformation of the Eye and Ear—Anophthalmos ... 163

CORRESPONDENCE ... 167

PROCEEDINGS OF SOCIETIES:

### *American Medical Association.*

The twentieth annual session will be held in New Orleans, La., May 4, 1869, at 11 A. M.

The following committees are expected to report:—On Diseases of the Cornea, Dr. Jos. S. Hildreth, Illinois, Chairman. On cultivation of the Cinchona Tree, Dr. Lemuel J. Deal, Pennsylvania, Chairman; On Excision of Joints for Injuries, Dr. J. B. Reed, Georgia, Chairman. On the Cryptogamic Origin of Disease with special reference to recent microscopic investigations on that subject, Dr. Edward Curtis, U. S. A., Chairman. On Operations for Hare-lip, Dr. A. Hammer, Missouri, Chairman. On Clinical Thermometry in Diptheria, Dr. Jos. G. Richardson,

New York, Chairman. On Prophylactics in Zymotic Diseases, Dr. Nelson L. North, New York, Chairman. On Inebriate Asylums, Dr. C. H. Nichols, D. C., Chairman. On the Influence of the Pneumogastric Nerve on Spasmodic and Rythmical Movements of the Lungs, Dr. Antisell, D. C., Chairman. To examine into the Present Plan of Organization and Management of the United States Marine Hospitals, Dr. D. W. Bliss, D. C. Chairman. On the Utilization of Sewerage, Dr. Stephen Smith, New York, Chairman. On the Influence of Quarantine in Preventing the Introduction of Disease into the ports of the United States, Dr. Elisha Harris, N. Y., Chairman. On Nurse Training Institutions, Dr. Samuel D. Gross, Pennsylvania, Chairman. On Commissioners to aid in Trials involving Scientific Testimony, Dr. John Ordronaux, N. Y., Chairman. On Annual Medical Register, Dr. John H. Packard, Pennsylvania, Chairman, On Devising a Plan for the Relief of Widows and Orphans of Medical Men, Dr. John H. Griscom, N. Y., Chairman. On Veterinary Colleges, Dr. Thomas Antisell, D. C., Chairman. On Specialties in Medicine, and the Propriety of Specialitists Advertising, Dr. E. Lloyd Howard, Maryland, Chairman. On Library of American Medical Works, Dr. J. M. Toner, D. C., Chairman. On Vaccination, Dr. Henry A. Martin, Massachusetts, Chairman. On the Decomposition of Urea in Uræmic Poisoning, Dr. H. R. Noel, Maryland, Chairman. On the best method of treatment for the different forms of Cleft Palate, Dr. J. R. Whitehead, New York, Chairman. On Rank of Medical Men in the Navy, Dr. N. S. Davis, Illinois, Chairman. On Medical Ethics, Dr. D. Francis Condie, Pennsylvania, Chairman. On American Medical Necrology, Dr. C. C. Cox, Maryland, Chairman. On Medical Education, Dr. J. C. Beeve, Ohio, Chairman. On Medical Literature, Dr. E. Warren, Maryland, Chairman. On Prize Essays, Dr. S. M. Bemiss, Lousiana, Chairman.

On the Climatology and Epidemics of——Maine, Dr. J. C. Weston. New Hampshire, Dr. P. A. Stackpole.

Vermont, Dr. Henry James. Massachusetts, Dr. H. I. Bowditch. Rhode Island, Dr. C. W. Parsons. Connecticut, Dr. E. K. Hunt. New York, Dr. W. F. Thoms. New Jersey, Dr. Ezra M. Hunt. Pennsylvania, Dr. D. F. Condie. Maryland, Dr. O. S. Mahon. Georgia, Dr. Juria Harris. Missouri, Dr. Geo. Engelman. Alabama, Dr. R. F. Michel. Texas, Dr. T. J. Heard. Illinois, Dr. R. C. Hamill. Indiana, Dr. J. F. Hibbard. District of Columbia, Dr. T. Antisell. Iowa, Dr. J. C. Hughes. Michigan, Dr. Abm. Sager. Ohio, Dr. E. L. Neal. California, Dr. F. W. Hatch. Tennessee, Dr. B. W. Avent. West Virginia, Dr. E. A. Hildreth. Minnesota, Dr. Samuel Willey. Virginia, Dr. W. O. Owen. Delaware, Dr. L. B. Bush. Arkansas, Dr. G. W. Lawrence. Mississippi, Dr. ——— Compton. Louisiana, Dr. L. T. Pimm.

Secretaries of all medical organizations are requested to forward lists of their Delegates as soon as elected, to the Permanent Secretary.

Any respectable physician who may desire to attend, but cannot do so as a delegate, may be made a *member by invitation*, upon the recommendation of the Committee of Arrangements. W. B. ATKINSON.

### *Delegates to the American Medical Association.*

The undersigned are fully authorized to announce that arrangements have been completed with the proper rail road officers, by which all delegates and members of the American Medical Association who desire to attend the annual meeting in New Orleans on the first Tuesday in May, can go from Chicage, by way of Louisville, Nashville, etc., direct to New Orleans, by paying the ordinary fare ($35.00) in going, and be returned *free*, with the privilege of stopping over at the Mammoth Cave, in Kentucky, if they choose. Similar arrangements are in progress, with a fair prospect of completion, with all the connecting lines of rail road at this place and southward. It is confidently expected that such liberal arrangements on the part of the rail road officers will secure a large attendance of members from the North and Northwest.

CHICAGO, March 3d, 1669.

N. S. DAVIS,

R. C. HAMILL, } Committee.

A. FISHER.

### *Illinois State Medical Society.*

The next annual meeting of the Illinois State Medical Society will be held in the city of Chicago, on the third Tuesday in May, 1869, commencing at 10 o'clock, A. M. All local medical societies are entitled to one delegate for every five members. It is desirable that every county in the State should be represented.

CHICAGO, March 3d, 1869.

N. S. DAVIS, *Perm't Secretary.*

